# SLC25A32 promotes malignant progression of glioblastoma by activating PI3K-AKT signaling pathway

**DOI:** 10.1186/s12885-023-11097-6

**Published:** 2023-06-26

**Authors:** Zhiwei Xue, Jiwei Wang, Zide Wang, Junzhi Liu, Jiangli Zhao, Xuchen Liu, Yan Zhang, Guowei Liu, Zhimin Zhao, Wenjie Li, Qing Zhang, Xingang Li, Bin Huang, Xinyu Wang

**Affiliations:** 1 Department of Neurosurgery, Qilu Hospital, Cheeloo College of Medicine, Institute of Brain and Brain-Inspired Science, Shandong University, Jinan, 250012 China; 2 Jinan Microecological Biomedicine Shandong Laboratory and Shandong Key Laboratory of Brain Function Remodeling, Jinan, 250117 China

**Keywords:** SLC25A32, PI3K-AKT, Folate, Metabolism, Glioblastoma

## Abstract

**Background:**

Solute carrier family 25 member 32 (SLC25A32) is an important member of SLC25A family and plays a role in folate transport metabolism. However, the mechanism and function of SLC25A32 in the progression of human glioblastoma (GBM) remain unclear.

**Methods:**

In this study, folate related gene analysis was performed to explore gene expression profiles in low-grade glioma (LGG) and GBM. Western blotting, real-time quantitative PCR (qRT-PCR), and immunohistochemistry (IHC) were used to confirm the expression levels of SLC25A32 in GBM tissues and cell lines. CCK-8 assays, colony formation assays, and Edu assays were performed to assess the role of SLC25A32 on proliferation in GBM in vitro. A 3D sphere invasion assay and an ex vivo co-culture invasion model were performed to assess the effects of SLC25A32 on invasion in GBM.

**Results:**

Elevated expression of SLC25A32 was observed in GBM, and high SLC25A32 expression was associated with a high glioma grade and poorer prognosis. Immunohistochemistry performed with anti-SLC25A32 on samples from an independent cohort of patients confirmed these results. Knockdown of SLC25A32 inhibited the proliferation and invasion of GBM cells, but overexpression of SLC25A32 significantly promoted cell growth and invasion. These effects were mainly due to the activation of the PI3K-AKT-mTOR signaling pathway.

**Conclusion:**

Our study demonstrated that SLC25A32 plays a significant role in promoting the malignant phenotype of GBM. Therefore, SLC25A32 can be used as an independent prognostic factor in patients with GBM, providing a new target for the comprehensive treatment of GBM.

**Supplementary Information:**

The online version contains supplementary material available at 10.1186/s12885-023-11097-6.

## Introduction

Glioblastoma (GBM) is currently one of the most common human primary malignant brain tumors [1, 2]. Despite an increased molecular understanding of GBM and advances in surgical techniques, radiotherapy and chemotherapy, the prognosis of GBM patients remains poor, [3, 4], with a 5-year relative survival rate of only 5% [1, 5]. Therefore, there is an urgent need to find new molecular biomarkers to improve the therapeutic effect of currently used drugs on glioblastoma and to explore new therapeutic strategies.

Cancer is characterized by reprogrammed energy metabolism, which plays a critical role in tumorigenesis, progression, and treatment resistance [6]. After Warburg discovered alterations in cancer metabolism, the advent of anti-folate metabolic therapy transformed the treatment regimen for childhood leukemia [7]. Over the past decade, however, there have been limited advances in treatments targeting the various metabolic pathways of cancer. Folate, also known as vitamin B9, is an essential nutrient that can be obtained from the diet because animals, including humans, do not have the biochemical capacity to resynthesize folate [8]. Folate metabolism regulates the bioavailability of folic acid and is an important component of carbon metabolism, which maintains cancer cell proliferation by producing one carbon unit for biosynthesis, epigenetic modification, and redox homeostasis [9], and glioblastoma is no exception. At present, the mechanisms of folate metabolism and carbon metabolism in the pathogenesis of glioblastoma are still poorly understood.

Solute carrier (SLC) proteins are a group of transmembrane transporters that mediate the inflow and outflow of solutes across the plasma membrane and intracellular membrane [10]. To date, approximately 300 SLC genes have been cloned and divided into 43 families. Each family of SLC vectors is capable of transporting specific substrates such as amino acids, oligopeptides, sugars, monocarboxylic acids, organic cations, anions, phosphates, nucleosides, metals, and water-soluble vitamins [11]. SLC25A members, the largest solute carrier group of the solute carrier family [12], link several metabolic processes among different cellular compartments (cytoplasmic matrix and mitochondrial matrix) by mediating solute translocation across the membrane [13]. SLC25A members appear to play multiple roles in tumorigenesis, including metabolic reprogramming, mitochondrial apoptosis, cell redox homeostasis, tumor proliferation and promotion of cancer stem cell stemness and chemoresistance [14–21]. Solute carrier family 25 member 32 (SLC25A32) is an important member of the SLC25A family and plays a role in folate transport metabolism [22]. SLC25A32 plays a role in tumor progression [23–25], but research on SLC25A32 in glioma is still lacking.

In this study, we confirmed that SLC25A32 is highly expressed in glioma compared to normal brain tissue. In addition, the expression level of high-grade gliomas was higher than that of low-grade gliomas. We further revealed that SLC25A32 supports the proliferation and invasion of GBM cells. Moreover, we demonstrated that SLC25A32 activates the PI3K-AKT signaling pathway in GBM cells to maintain malignant phenotype, suggesting that SLC25A32 is a potential therapeutic target in GBM.

## Methods

### Database searches

The Cancer Genome Atlas (TCGA, http://cancergenome.nih.gov), Chinese Gliomas Genome Atlas (CGGA, http://www.cgga.org.cn/), Rembrandt (http://www.betastasis.com/glioma/rembrandt), and folate related gene sets were downloaded from the GSEA website (http://www.broadinstitute.org/gsea/). The Human Protein Atlas (http://www.proteinatlas.org) was mined for relevant molecular data. Heatmaps were generated using the function ‘heatmap.2’ from the R package ‘gplots v3.0.1’ (https://CRAN.R-project.org/package=gplots). Venn analysis was performed using the OmicStudio tools at https://www.omicstudio.cn/tool. Enrichment of GO terms and KEGG pathways was performed in R version 4.2.1. SLC25A32 related genes were found in Linked Omics (http://linkedomics.org/) database. Kaplan-Meier survival curves were generated and compared using the log-rank test. P values determined from different comparisons < 0.05 were considered statistically significant and are indicated as follows: *P < 0.05; ** P < 0.01; *** P < 0.001. Univariate analyses of OS were performed using Cox regression and presented by forest using the R version 4.2.1. A nomogram model was constructed based on the expression of SLC25A32 and other clinical features (grade, gender, age, radio status, chemotherapy status, IDH mutation status and 1p19q co-deletion status). The “rms” package in R software was used to calculate the score, and the 1, 3 and 5 year survival of glioma patients was predicted based on the total score.

### Patients and samples

Glioma specimens and adjacent normal tissues were obtained from Qilu Hospital of Shandong University (Jinan, China). This study protocol was approved by the Scientific Research Ethics Committee of Qilu Hospital, Shandong University (approval number: KYLL-2017(KS)-090). For their engagement in this study, patients and volunteers gave written informed consent. Samples of human glioma tissue were taken during surgery on patients at the Qilu Hospital. Due to traumatic brain injury occurrences, non-neoplastic brain tissue samples were collected from patients during surgery. Glioma specimens were validated and classified by two experienced clinical pathologists according to the WHO tumor classification.

#### Cell lines and cultures

The human glioma cell line LN229 was purchased from the Chinese Academy of Sciences Cell Bank (Shanghai, China). GBM#P3, GBM#BG5 and luciferase-stable GBM#P3 were established from GBM surgical specimens at the K. G. Jebsen Brain Tumor Research Centre, Department of Biomedicine, University of Bergen (Bergen, Norway) and kindly provided by Prof. Rolf Bjerkvig. LN229 cells were cultured in Dulbecco modified Eagle medium (DMEM; Thermo Fisher Scientific, SH30022.01B) supplemented with 10% fetal bovine serum (FBS, GE Healthcare Life Sciences, 10,082,147). GBM#P3 and GBM#BG5 cells were cultured in Neurobasal Medium (Thermo Fisher Scientific; Waltham, MA, USA) containing B27 supplement (20 µL/mL), FGF (20 ng/mL) and EGF (20 ng/mL) for cell culture and passage. The culture medium of GBM#P3 and GBM#BG5 cells was replaced with DMEM supplemented with 10% FBS for cell plating and further functional experiments. Cells were maintained at 37 °C in a humidified chamber containing 5% CO2.

### SLC25A32 silencing and overexpression

Small interfering RNAs (siRNAs) targeting SLC25A32 were requested from BioSune (Shanghai, China) to silencing SLC25A32. The sequence of the siRNAs directed against SLC25A32 was as follows.: si-SLC25A32#1: GCUGCCAUCAACACAAUGUTT, si-SLC25A32#2: GCAGAUGAAUGCUUUAUAATT. For overexpression of SLC25A32 in GBM cells, the full-length SLC25A32 cDNA was inserted into the pENTER vector. Small interfering RNA (siRNA) transfection was performed using Lipofectamine™ 2000 (Invitrogen). The plasmid DNA was transfected using a DNA transfection reagent (Lipofectamine lipo3000; Invitrogen) according to the manufacturer’s instruction. Following 48 h of transfection, the cells were harvested, and qRT-PCR, western blot analysis, and other tests were performed to validate the knockdown or overexpression efficiency of the cells.

### Quantitative real-time PCR (qRT-PCR)

Following the manufacturer’s instructions, total RNA was extracted using a kit from Yishan Bio, China. cDNA was produced using the ReverTra Ace qPCR RT Kit from whole RNA (TOYOBO; Osaka, Japan). SYBR Green Master Mix (Roche; Basel, Switzerland) was used to run qRT-PCR on the 480II Real Time PCR Detection System (Roche; Basel, Switzerland). The expression of mRNA was standardized using GAPDH mRNA. The results are representative of at least three independent experiments. The sequences of the PCR primers used are the follows: GAPDH-F 5’-GCACCGTCAAGGCTGAGAAC-3’, R 5’- TGGTGAAGACGCCAGTGGA-3’; SLC25A32-F 5’-GCCCAGTTGAGCACAGTAGA-3’, R 5’-AAAATCCACCGACGCCTTCT-3’.

### Western blot analysis

Western blot analysis of cell lysates (20 µg protein) was performed in accordance with previously mentioned techniques. The aforementioned antibodies were used to incubate membranes: SLC25A32 (Proteintech, 13080-1-AP), GAPDH (Cell Signaling Technology, 5174 S), Phospho-Akt (Ser473)) (Cell Signaling Technology, 4060 S), AKT (Cell Signaling Technology, 4691 S). Phospho-mTOR (Ser2448) (Cell Signaling Technology, 5536T), mTOR (Proteintech, 66888-1-Ig).

#### Cell proliferation assay

By using the Cell Counting Kit-8 (CCK8) assay, colony formation assay, and EdU Assay, the capacity of cells to proliferate was determined. The cells were treated with CCK-8 (10 µl/well) for 2 h after being seeded as 3 × 10^3^ cells suspension in 96-well plates. The optical density value (OD value) was then measured at 450 nm.

A 6-well plate with 1000 infected LN229 cells was utilized for the colony formation experiment. After 14 days of incubation, the surviving cell colonies were fixed, stained with crystal violet, and the colony counts were calculated using ImageJ.

The standard procedure provided by the manufacturer was followed while using the EdU (RiboBio, Guangzhou, China) assay. ImageJ was used to calculate the outcomes. At least three times each experiment was run repeatedly.

### Tumor sphere formation assay

For the tumor sphere formation assay, 1000 GSCs with SLC25A32 knockdown or overexpression were plated into each well of a 96-well plate, and the tumor sphere number and diameter was calculated on the sixth day after cell seeding.

### Cell invasion assays

For Transwell analysis, Matrigel (BD Biosciences, NJ) was used to cover the bottom membrane of Transwell chambers (24 holes, Corning Inc., NY) and the invasive ability of the cells was measured. Each Transwell membrane contained 50 µl of a mixture of Matrigel and medium at a proportion of 1:2. The upper chamber was inoculated with 3 × 10^4^ LN229 cells with SLC25A32 knockdown, in medium without FBS, and medium containing 30% FBS was placed in the lower chamber, and cultured in 5% CO_2_ at 37 °C for 36 h. After stabilizing the chambers for 10 min with paraformaldehyde, 500 µL of 0.1% crystal violet was added for 10 min before being removed. The stained cells were counted and photographed using a light microscope (200×) after being allowed to air dry in four randomly chosen areas.

GBM#P3 or GBM#BG5 cells (3 × 10^3^/well) with SLC25A32 knockdown or overexpression were seeded onto poor adherence 96-well plates for 3D tumor sphere invasion tests. Invasion gel (50 µl/well; R&D Systems;3500-096-03; Minneapolis, MN, USA) was added to the wells after the creation of the spheres, and the plates were incubated for 72 h. To evaluate the capacity of modified tumor cells to invade, images were taken at predetermined intervals.

To measure GBM cell invasion, in vitro GBM brain organoid co-culture procedures, such as the cultivation of 28-day rat embryonic brain organoids, were also performed. GFP-transfected GBM cells were first co-cultivated for 48 h with mature brain organoids after being cultured to facilitate the creation of spheres. To evaluate the capacity to invade, confocal microscopy images of co-cultures were collected (Leica TCS Sp8; Wetzlar, Germany).

### Immunohistochemistry

Samples that had been paraffin-embedded were cut into 4 μm sections and placed on microscope slides. In a microwave, heat-induced epitope retrieval was carried out in a pH 7.2 citric acid buffer solution. After rinsing with PBS and being coated with a secondary antibody made of goat anti-rabbit and horseradish peroxidase, sections were incubated with the primary antibody SLC25A32 (Proteintech, 13080-1-AP) overnight at 4 °C (ZSGB-BIO, PV-9000). Slides were counterstained with Mayer haematoxylin after diaminobenzidine (ZSGB-BIO, ZLI-9033) was used as the substrate to enable visualization (Beyotime Biotechnology, C0107).

### Statistical analysis

The results from three separate studies were represented as the mean minus the standard deviation (SD). In the GraphPad Prism 8 program, data were compared using unpaired Student’s t test for two-group comparisons and one-way analysis of variance (ANOVA) for multigroup comparisons (San Diego, CA, USA). The following are the P values from several comparisons that were deemed statistically significant and are indicated: *P < 0.05; ** P < 0.01; *** P < 0.001.

## Results

### Identification of differentially expressed folate metabolism-related genes (DEGs) in glioma

**Fig. 1:**
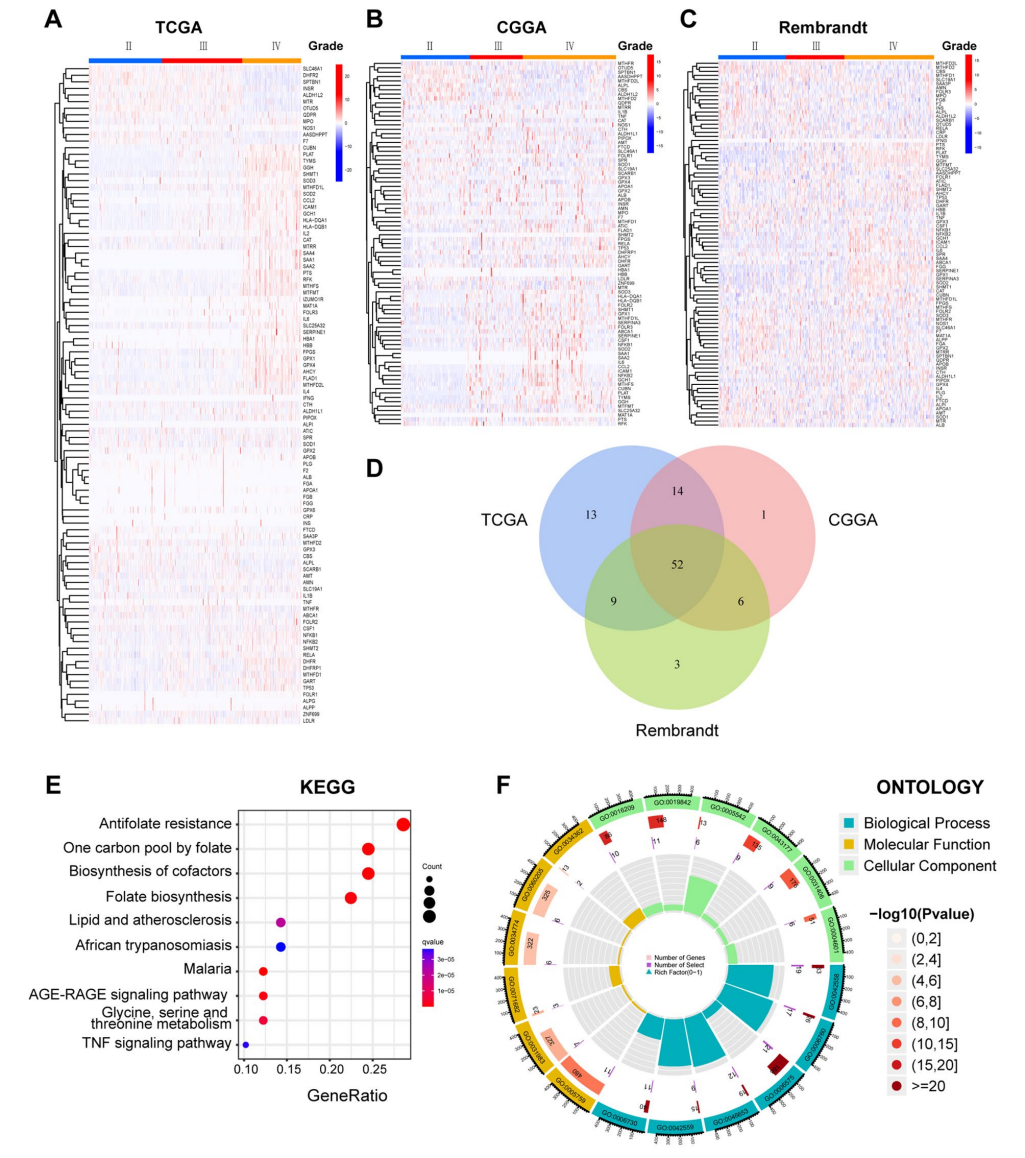
**Differentially expressed genes and common differentially expressed genes in three datasets** (**A**) The different expressed folate related genes in different grade of glioma in TCGA; (**B**) The different expressed folate related genes in different grade of glioma in CGGA; (**C**) The different expressed folate related genes in different grade of glioma in Rembrandt; (**D**) Commonly upregulated genes in three datasets, the various colored areas show different datasets; (**E**) Analysis of gene enrichment of the KEGG pathway for different expressed genes; (**F**) GO enrichment for different expressed genes

To identify the effect of folate metabolism-related genes in glioma, 104 folate metabolism-related genes were obtained from GSEA gene sets. Folate metabolism-related gene expression profiles were collected from three public databases: TCGA, CGGA, and Rembrandt. The gene expression profiling was analyzed by the “limma” and “heatmap” packages of R software version 4.2.1 [26]. The DEGs of the three datasets are shown as heatmap (Fig. 1A, B, C). The intersection of the Venn diagram revealed 52 common upregulated genes (Fig. 1D). To explore the potential role of these 52 up-regulated genes in glioma progression, KEGG and GO enrichment analyses were performed (Fig. 1E, F). Not surprisingly, both KEGG and GO enrichment analyses revealed that the genes were associated with folate and one carbon unit metabolism related pathways, indicating that the gene set we selected was closely related to folate metabolism. SLC25A32, an SLC family molecule closely related to folic acid transport, was screened out from the 52 DEGs for further exploration.

### Correlation between SLC25A32 expression level and glioma clinical features

**Fig. 2:**
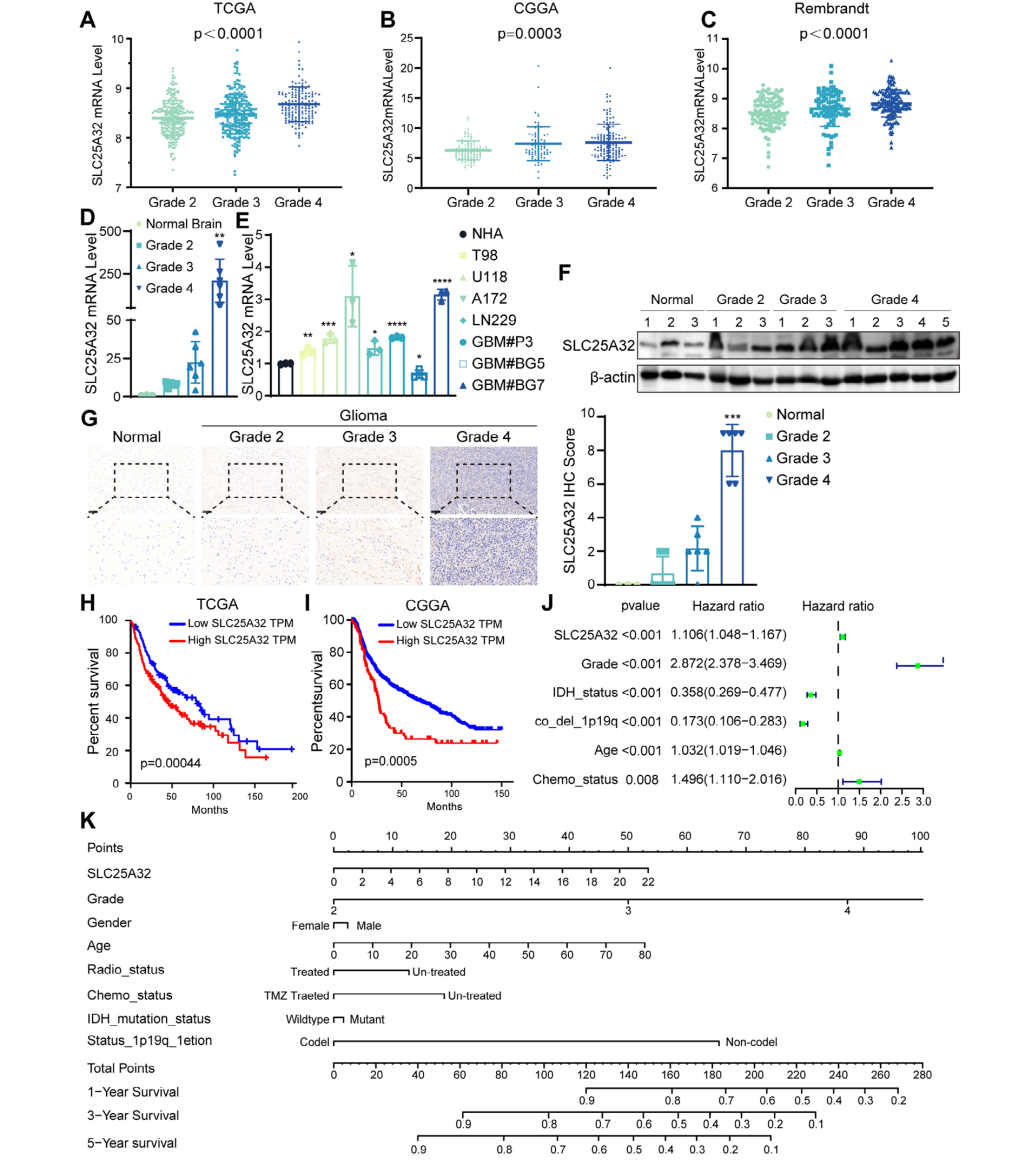
**SLC25A32 is elevated in glioma and associated with poor prognosis in glioma patients** (**A**) The expression level of SLC25A32 in different grade glioma patient in TCGA-LGG GBM; (**B**) The expression level of SLC25A32 in different grade glioma patient in CGGA; (**C**) The expression level of SLC25A32 in different grade glioma patient in Rembrandt; (**D**) RNA expression levels of SLC25A32 in different grade human glioma and normal brain tissue (NBT) samples; (**E**) RNA expression levels of SLC25A32 in normal human astrocyte and glioma cell lines T98, U118, A172, LN229, GBM#P3, GBM#BG5, GBM#BG7; (**F**) Protein expression levels of SLC25A32 in different grade human glioma and normal brain tissue (NBT) samples; (**G**) Representative immunostaining and quantification of SLC25A32 in different grade human glioma and normal brain tissue (NBT) samples (magnification: × 200, × 400) (scale bars: 100 μm); (**H**) Kaplan-Meier curves for SLC25A32 in glioma cohort in TCGA; (**I**) Kaplan-Meier curves for SLC25A32 in glioma cohort in CGGA; (**J**) Univariate analyses for predictors of overall survival in the CGGA dataset; (**K**) The nomogram plot was built based on the expression of SLC25A32, Grade, Gender, age, Radio status chemotherapy status, IDH status and 1p19q co deletion status in glioma. Then directly convert total points to particular 1-year, 3–year, and 5–year related survival probabilities

The expression levels of SLC25A32 were first analyzed in TCGA-GBM LGG (n = 669), CGGA (n = 321), and Rembrandt (n = 555) (Fig. 2A, B, C). We found that SLC25A32 expression was positively correlated with increasing grade. The RNA expression levels of SLC25A32 were also consistent with the above results in our own glioma cohort (Fig. 2D). The RNA level of SLC25A32 in glioma cell lines was also higher than that in normal astrocytes (Fig. 2E). To verify whether SLC25A32 protein levels increased with glioma grade, western-blot was performed, and results showed that SLC25A32 protein levels also higher than that in normal brain tissues. Moreover, SLC25A32 protein expression level increased with the increase of glioma grade (Fig. 2F). SLC25A32 protein expression levels were collected from the Human Protein Atlas (Supplementary Fig. 1A). SLC25A32 immunohistochemistry (IHC) was performed on glioma (n = 18) and normal brain tissue samples (n = 3) collected from Qilu Hospital. The results showed that SLC25A32 was low in normal brain tissue, but increased with the increasing of glioma grade (Fig. 2G). In addition, Kaplan-Meier survival analysis demonstrated that LGG and GBM patients with high SLC25A32 expression also had poor overall survival outcomes (Fig. 2H, I). Through univariate Cox regression analysis of CGGA gene expression profile, the effects of the SLC25A32 expression level and other clinical characteristics on the survival of glioma patients were found. The results showed that grade, age, chemotherapy status and SLC25A32 expression level were independent prognostic factors affecting overall survival (OS). (Fig. 2J). As a result, a nomogram was created to forecast the likelihood that glioma patients will survive, integrated with the important prognostic markers (Fig. 2K). The total scores were immediately transformed into specific 1-year, 3-year, and 5-year linked survival chances by summing up the whole score and placing it on the total point scale. The findings above indicated that increased SLC25A32 expression was connected to higher grade gliomas and could serve as a prognostic indicator for GBM patients.

### SLC25A32 promotes GBM cell proliferation

**Fig. 3:**
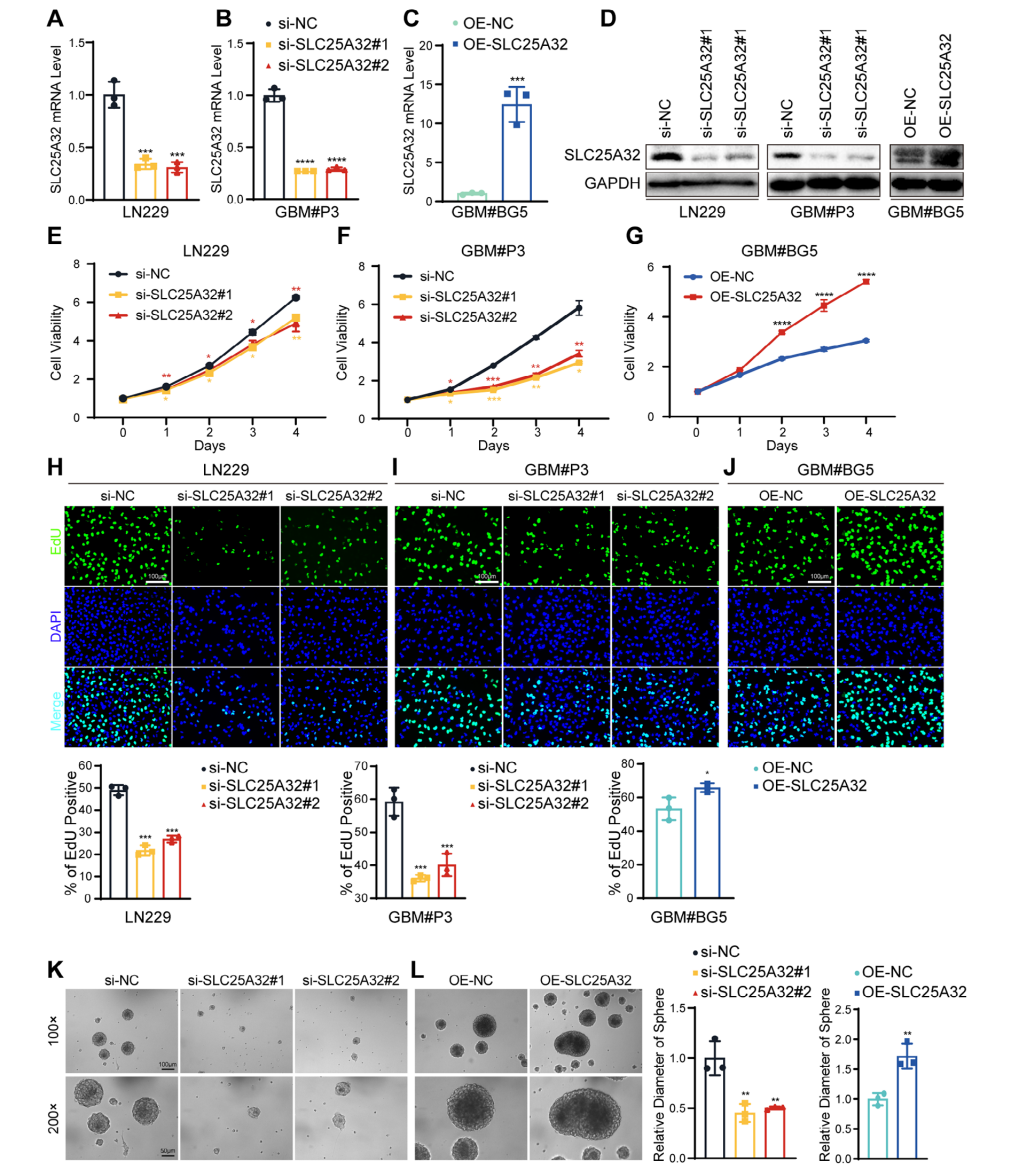
**SLC25A32 promotes GBM cells proliferation** (**A-C**) qRT-PCR was used to verify the knockdown or overexpress efficiency of SLC25A32 in GBM cells; (**D**) Western-blot was used to verify the knockdown or overexpress efficiency of SLC25A32 in GBM cells; (**E**) CCK8 assays showing the proliferation ability of GBM cells LN229 transfected with si-NC or si-SLC25A32; (**F**) CCK8 assays showing the proliferation ability of GBM cells GBM#P3 transfected with si-NC or si-SLC25A32; (**G**) CCK8 assays showing the proliferation ability of GBM cells GBM#BG5 transfected with OE-NC or OE-SLC25A32; (**H**) Fluorescence images and quantification of EdU assays performed to assess the proliferative capacity of GBM cells LN229 transfected with si-NC or si-SLC25A32 (scale bar, 100 μm); (**I**) Fluorescence images and quantification of EdU assays performed to assess the proliferative capacity of GBM cells GBM#P3 transfected with si-NC or si-SLC25A32 (scale bar, 100 μm); (**J**) Fluorescence images and quantification of EdU assays performed to assess the proliferative capacity of GBM cells GBM#BG5 transfected with OE-NC or OE-SLC25A32 (scale bar, 100 μm); (**K**) Representative tumor sphere images of GBM cells GBM#P3 transfected with si-NC or si-SLC25A32 (scale bar, 100 μm); (**L**) Representative tumor sphere images of GBM cells GBM#BG5 transfected with OE-NC or OE-SLC25A32 (scale bar, 100 μm)

To investigate the biological role and underlying mechanism of SLC25A32 in GBM, LN229 and GBM#P3 cells with relatively higher expression of SLC25A32 were transfected with two independent siRNAs or negative control, and knockdown efficiency of the siRNAs was confirmed by qRT-PCR and western-blotting (Fig. 3A, B, D). GBM#BG5 with lower expression of SLC25A32 were transfected with wild-type (WT) full-length SLC25A32 vectors or the empty vector control. The efficiency of overexpression was confirmed by qRT-PCR and western-blotting (Fig. 3C, D).

The CCK-8 and colony formation assays were used to investigate how SLC25A32 affected growth characteristics. The results revealed that SLC25A32 knockdown significantly reduced LN229 and GBM#P3 cell growth. (Fig. 3E, F) and colony formation of LN229 cells (Supplementary Fig. 1B), whereas overexpression of SLC25A32 significantly promoted the growth of GBM#BG5 cells (Fig. 3G, Supplementary Fig. 1C). EdU assays further confirmed the proliferation-promoting effect of SLC25A32 on GBM cell lines (Fig. 3H-J). We conducted a spheroid formation assay to evaluate the ability of GSCs to self-renew to determine whether SLC25A32 plays a significant role in stemness and self-renewal. (Fig. 3K, L, Supplementary Fig. 1D). The quantitative study revealed that SLC25A32 overexpression significantly increased the size and effectiveness of GBM#BG5 cell spheroid formation. In addition, endogenous SLC25A32 knockdown decreased the size and effectiveness of neural sphere formation.

### SLC25A32 increases GBM cell invasive characteristics

**Fig. 4:**
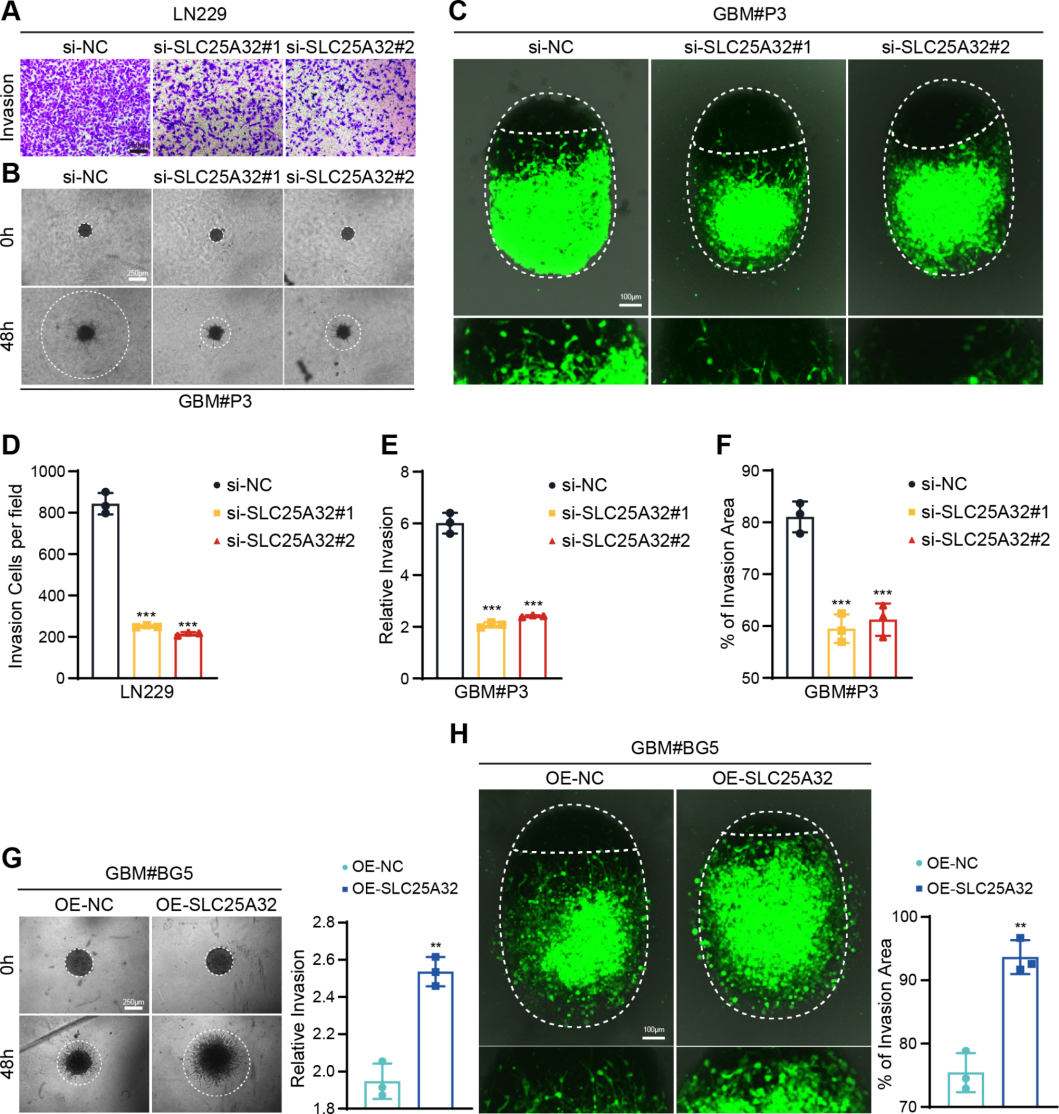
**SLC25A32 Promotes the invasive properties of GBM cells** (**A, D**) Representative images and quantification of Transwell assays performed on GBM cells LN229 transfected with si-NC or si-SLC25A32 (scale bar, 250 μm); (**B, E**) Representative images of 3D tumor sphere invasion assays and quantification for GBM cells GBM#P3 transfected with si-NC or si-SLC25A32. Representative images were acquired at 48 h, and plots show percentage of invaded area (scale bar, 250 μm); (**C, F**) Model and representative fluorescence images of co-culture invasion assays of GBM cells GBM#P3 transfected with si-NC or si-SLC25A32. Invasion capacity was assessed at 72 h (scale bar, 100 μm); (**G**) Representative images of 3D tumor sphere invasion assays and quantification for GBM cells GBM#BG5 transfected with OE-NC or OE-SLC25A32. Representative images were acquired at 48 h, and plots show percentage of invaded area (scale bar, 250 μm); (**H**) Model and representative fluorescence images of co-culture invasion assays of GBM cells GBM#BG5 transfected with OE-NC or OE-SLC25A32. Invasion capacity was assessed at 72 h (scale bar, 100 μm)

We examined whether SLC25A32 overexpression or knockdown affected the ability of GBM cells to invade. First, we investigated the invasive capacity of altered LN229 cells in the Transwell migration experiment, and observed that the percentage of cells that crossed the cell membrane in the siRNA group was much lower than that in the control group. (Fig. 4A, D). Then, using a 3D sphere invasion experiment, we examined the invasive capabilities of GBM#P3 cells. Compared to the control cell population, the relative invasion of GBM#P3-siSLC25A32 spheroids was less. (Fig. 4B, E). We next added GBM#P3-siSLC25A32 cells to a previously established ex vivo co-culture invasion model of tumor spheroids with normal rat brain-like organoids in order to more accurately simulate the physiologically aggressive tumor microenvironment [27]. GBM#P3- siSLC25A32 tumor spheroids had less invasive potential in this ex vivo model when compared to the control cell population (Fig. 4C, F). In contrast, the 3D sphere invasion test and the ex vivo co-culture invasion model showed that overexpressing SLC25A32 increased the invasion of GBM#BG5 cells (Fig. 4G, H).

### Pathway analysis of SLC25A32 and co-regulated genes in GBM

**Fig. 5:**
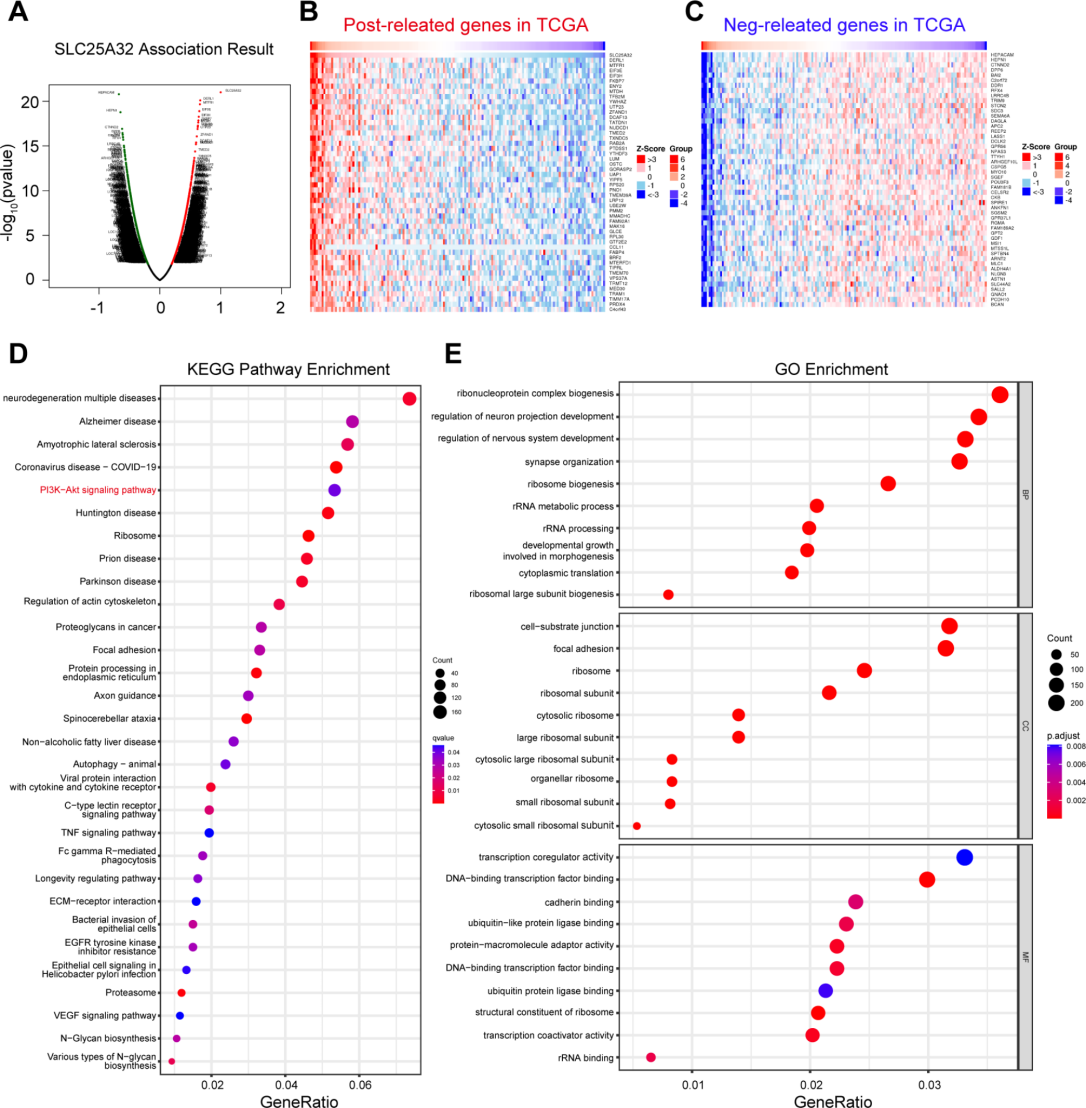
**Pathway analysis of SLC25A32 and co-regulated genes in GBM** (**A**) Volcano plot of genes correlated with SLC25A32 expression in the TCGA database; (**B**) Correlation analysis using TCGA data revealing positive correlated genes with SLC25A32 mRNA expression in human gliomas; (**C**) Correlation analysis using TCGA data revealing negative correlated genes with SLC25A32 mRNA expression in human gliomas; (**D**) KEGG pathway analysis [28–30] of the positive and negatively correlated genes of SLC25A32 in the TCGA database is illustrated; (**E**) GO enrichment analysis of the positive and negatively correlated genes of SLC25A32 in the TCGA database is illustrated

Correlation analysis of SLC25A32 in whole-genome gene profiling was performed by using the TCGA database to further understand the mechanism of SLC25A32 in GBM. Significant genes positively and negatively correlated with SLC25A32 are represented in the volcano plot (Fig. 5A) and heatmaps (Fig. 5B-C). Subsequently, KEGG enrichment analysis [28–30] revealed that SLC25A32-correlated genes were enriched in several important pathways and biological processes, such as PI3K-AKT signaling pathways (Fig. 5D). Biological activities such as cell-substrate junction, focal adhesion, ribonucleoprotein complex biogenesis, and transcription coregulator activity were substantially connected with SLC25A32 positively-correlated genes in GO analysis (Fig. 5E).

### SLC25A32 promoted the GBM malignant phenotype through the PI3K-AKT signaling pathway

**Fig. 6:**
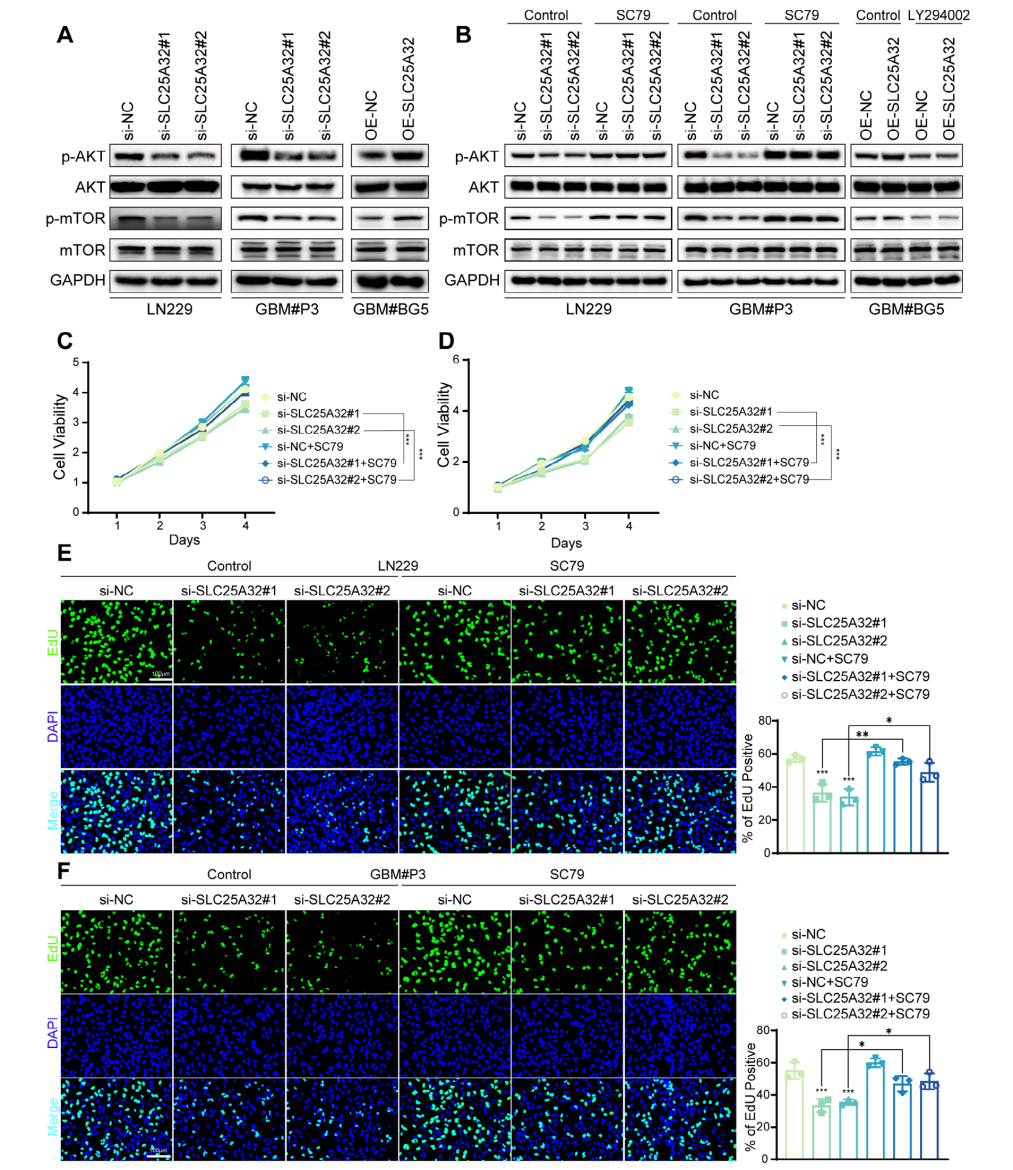
**SLC25A32 promoted GBM cells proliferation through PI3K-AKT signaling pathway** (**A**) Western blot analysis of the proteins level of p-AKT and AKT, p-mTOR and mTOR, GAPDH as the control in GBM cells LN229, GBM#P3 transfected with si-NC or si-SLC25A32 and GBM#BG5 transfected with OE-NC or OE-SLC25A32; (**B**) Western blot analysis of the proteins level of p-AKT and AKT, p-mTOR and mTOR, GAPDH as the control in GBM cells LN229, GBM#P3 transfected with si-NC or si-SLC25A32 or treated with SC79 and GBM#BG5 transfected with OE-NC or OE-SLC25A32 or treated with LY294002; (**C**) CCK8 assays showing the proliferation ability of GBM cells LN229 transfected with si-NC or si-SLC25A32 or treated with SC79; (**D**) CCK8 assays showing the proliferation ability of GBM cells GBM#P3 transfected with si-NC or si-SLC25A32 or treated with SC79; (**E**) Fluorescence images and quantification of EdU assays performed to assess the proliferative capacity of GBM cells LN229 transfected with si-NC or si-SLC25A32 or treated with SC79 (scale bar, 100 μm); (**F**) Fluorescence images and quantification of EdU assays performed to assess the proliferative capacity of GBM cells GBM#P3 transfected with si-NC or si-SLC25A32 or treated with SC79 (scale bar, 100 μm)

**Fig. 7:**
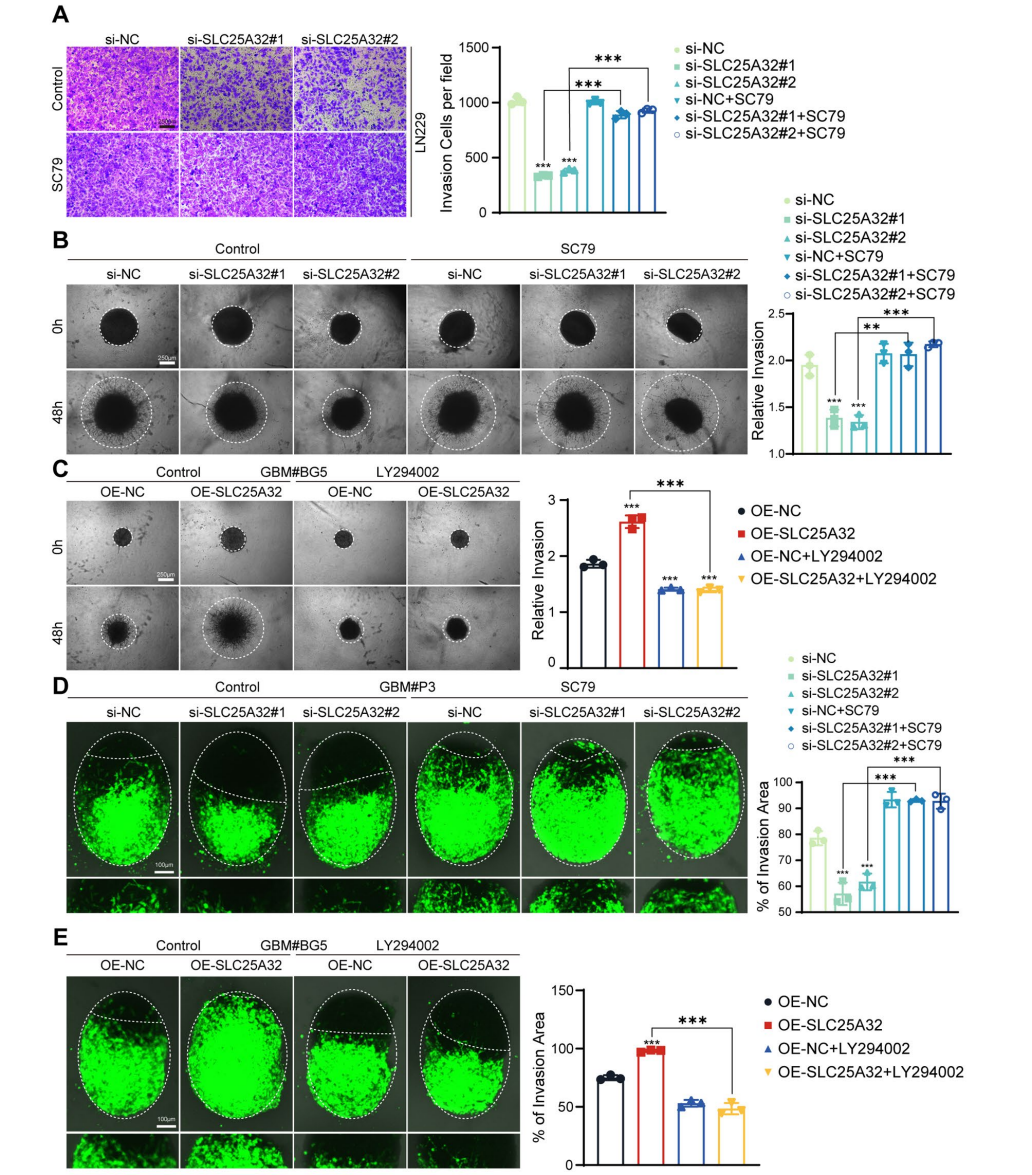
**SLC25A32 promoted GBM cells invasion through PI3K-AKT signaling pathway** (**A**) Representative images and quantification of Transwell assays performed on GBM cells LN229 transfected with si-NC or si-SLC25A32 or treated with SC79 (scale bar, 250 μm); (**B**) Representative images of 3D tumor sphere invasion assays and quantification for GBM cells GBM#P3 transfected with si-NC or si-SLC25A32 or treated with SC79. Representative images were acquired at 48 h, and plots show percentage of invaded area (scale bar, 250 μm); (**C**) Representative images of 3D tumor sphere invasion assays and quantification for GBM cells GBM#BG5 transfected with OE-NC or OE-SLC25A32 or treated with LY294002. Representative images were acquired at 48 h, and plots show percentage of invaded area (scale bar, 250 μm); (**D**) Model and representative fluorescence images of co-culture invasion assays of GBM cells GBM#P3 transfected with si-NC or si-SLC25A32 or treated with SC79. Invasion capacity was assessed at 72 h (scale bar, 100 μm); (**E**) Model and representative fluorescence images of co-culture invasion assays of GBM cells GBM#BG5 transfected with OE-NC or OE-SLC25A32 or treated with LY294002. Invasion capacity was assessed at 72 h (scale bar, 100 μm)

According to KEGG analysis, SLC25A32-correlated genes were enriched in the PI3K-AKT signaling pathway. It is widely known that aberrant activation of the PI3K-AKT-mTOR signaling pathway increases the malignant phenotype of malignancies. Therefore, we examined how ectopic SLC25A32 expression affected the expression of p-AKT (Ser473), which is essential for the activity and function of AKT. As shown in Fig. 6A, overexpression of SLC25A32 increased p-AKT (Ser473) and p-mTOR (Ser2448) protein expression in GBM#BG5 cells (Fig. 6A), whereas knockdown SLC25A32 decreased p-AKT (Ser473) and p-mTOR (Ser2448) protein expression in both LN229 and GBM#P3 cells (Fig. 6A). Using SC79, a specific AKT activator, to counteract the stimulation of the PI3K/AKT pathway and siSLC25A32-induced AKT inactivation, we further assessed the significance of PI3K-AKT signaling on SLC25A32-induced cell proliferation and invasion. (Fig. 6B). When co-treated with SC79 (10 µg/ml), the inhibition of GBM cell growth caused by SLC25A32 knockdown was rescued as proven by CCK8 and Edu assays (Fig. 6C-F). AKT activation caused by SLC25A32 and the stimulation of the PI3K-AKT pathway were both prevented by the administration of the PI3K inhibitor LY294002. In GBM cells, cotreatment with LY294002 (10 mM) dramatically reduced p-AKT and p-mTOR. (Fig. 6B). Consistently, SLC25A32-induced GBM#BG5 cell proliferation were reversed by targeted inhibition using LY294002 (Supplementary Fig. 1E, F). We then confirmed that SC79 could rescue GBM invasion decline caused by SLC25A32 knockdown by Transwell assay, 3D sphere invasion assay and ex vivo co-culture invasion model (Fig. 7A, B, D), indicating that SLC25A32 also increased GBM invasion through the PI3K-AKT-mTOR signaling pathway. Consequently, LY294002 abolished the effect of SLC25A32 overexpression on GBM#BG5 cell invasion (Fig. 7C, E). Overall, our results showed that SLC25A32 stimulates PI3K/AKT pathway activation, which leads to GBM cell proliferation and invasion.

## Discussion

A recent study have shown that SLC25A32, a member of the solute carrier 25 family of mitochondrial transporters [31], is a tetrahydrofolate (THF) transporter that can move tetrahydrofolate (THF) from the cellular cytoplasm to mitochondria in a test tube [32]. Tetrahydrofolate (THF) is the main form of folic acid that acts in the body, playing a crucial role as a carbon unit transporter in many reactions, which includes participating in the biosynthesis of DNA, decreasing plasma homocysteine levels and the biosynthesis of NO. Existing research has shown that the reduction or mutation of SLC25A32 may induce multiple acyl-CoA dehydrogenase deficiency, human myelomeningocele, MADD and related deficiencies, riboflavin-responsive exercise intolerance, neural tube defects and other diseases [22, 33–35]. Meanwhile, the reduction or mutation of SLC25A32 affects the progression of the tumor [23, 24]. Nevertheless, the biological roles and regulatory mechanisms of SLC25A32 in GBM are still not completely known.

In this study, we demonstrated that SLC25A32 expression is higher in GBM than in LGG, according to the data from public databases and IHC staining results. Moreover, the upregulation of SLC25A32 correlated with tumor histological grades in glioma and poorer prognosis. Our results also showed that knockdown of SLC25A32 markedly decreased proliferation and invasion ability of GBM cells. These data suggested that SLC25A32 aids the malignant phenotype of GBM cells. Based on these findings, SLC25A32 could be identified as a potential target of GBM molecular-targeting therapy.

SLC25A32 increases AKT and mTOR phosphorylation, which aids in the promotion of GBM malignant phenotypes. The main component of the PI3K-AKT-mTOR signaling pathway, AKT is a serine/threonine-specific protein kinase that is crucial for a number of cell growth activities, including glucose metabolism, apoptosis, cell proliferation, transcription, and cell migration. LY294002 is a PI3K inhibitor, that can inhibit the activation of PI3K-AKT-mTOR signaling pathway. Our experimental results showed that LY294002 inhibited the proliferation and invasion of tumor cells caused by SLC25A32. Additionally, p-AKT and p-mTOR levels as well as functional activities including proliferation, migration, and invasion were partially recovered in the presence of an AKT activator, SC79. In conclusion, the molecular foundation for the reduction of tumor development by SLC25A32 knockdown is through the PI3K-AKT-mTOR signaling pathway. Further research is necessary to determine the specific molecular pathways associated with the interaction of PI3K-AKT-mTOR signaling and SLC25A32 in GBM.

In conclusion, a series of bioinformatics analyses were performed to thoroughly investigate the genes involved in the advancement of GBM folate metabolic processes. We demonstrated that SLC25A32 is an oncogene in GBM, that induces activation of the PI3K-AKT-mTOR signaling pathway to promote the GBM malignant phenotypes. These results suggest that SLC25A32 could be used as a diagnostic biomarker and potential therapeutic target for GBM.

## Electronic supplementary material

Below is the link to the electronic supplementary material.

Supplementary Material 1

Supplementary Material 2

## Data Availability

Datasets and other files generated, analyzed, or used during this study are available from the corresponding author upon reasonable request.

## Abbreviations

DMEM: Dulbecco’s modified Eagle’s medium
GAPDH: Glyceraldehyde-3-phosphate dehydrogenase
GBM: Glioblastoma multiforme
GFP: Green fluorescent protein
FBS: Fetal bovine serum
EGF: Epidermal growth factor
FGF: Fibroblast growth factor
