# Supplementary figures and images for: SLC25A32 promotes malignant progression of glioblastoma by activating PI3K-AKT signaling pathway
Source: BMC Cancer. 2023 Jun 26;23:589. doi: 10.1186/s12885-023-11097-6 (PMC10294537; doi:10.1186/s12885-023-11097-6)

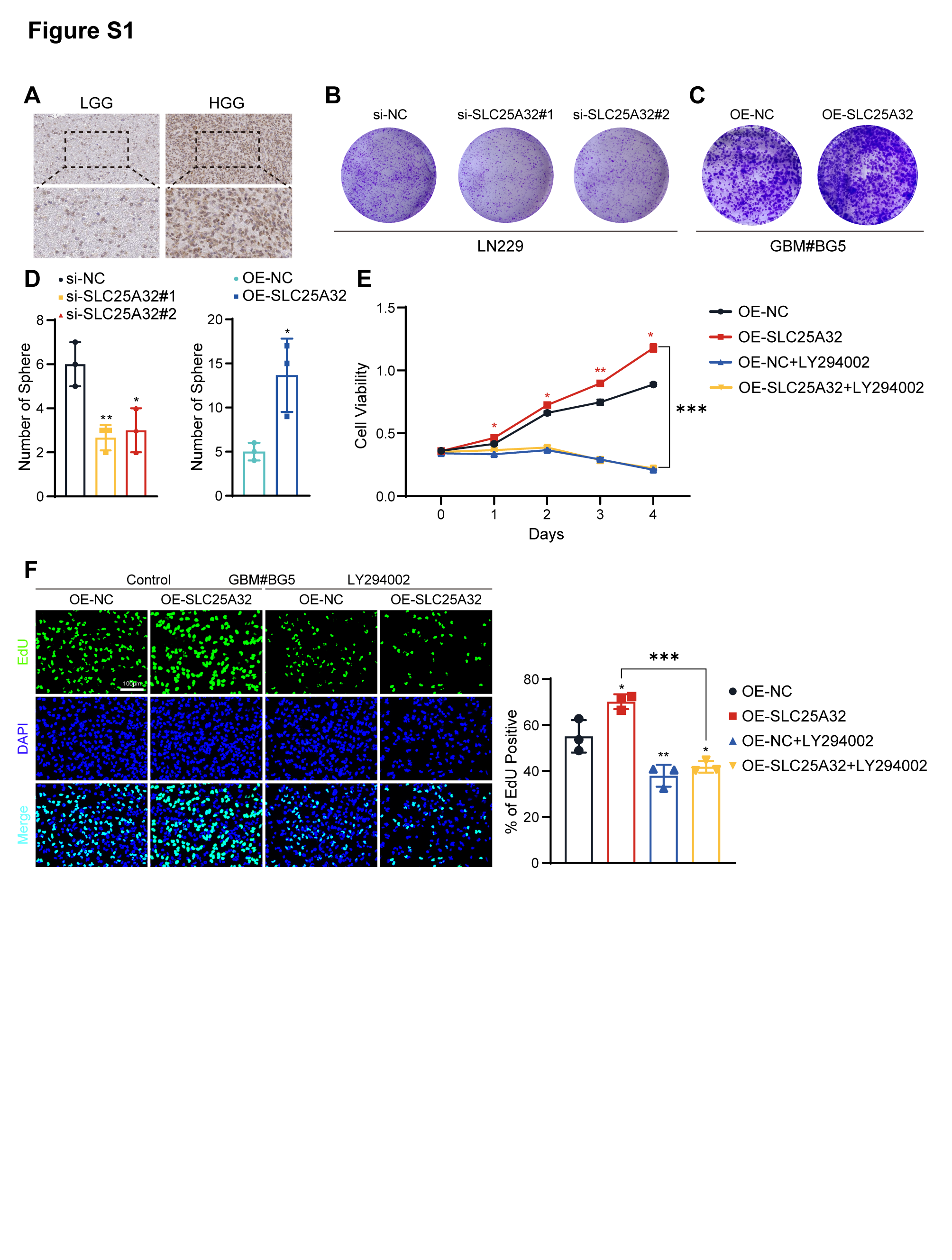

Supplement: Supplementary file 1 — Supplementary Material 1 [file 12885_2023_11097_MOESM1_ESM.tif]
