# Supplementary material for: SLC25A32 promotes malignant progression of glioblastoma by activating PI3K-AKT signaling pathway
Source: BMC Cancer. 2023 Jun 26;23:589. doi: 10.1186/s12885-023-11097-6 (PMC10294537; doi:10.1186/s12885-023-11097-6)
Supplement: Supplementary file 2 — Supplementary Material 2 [file 12885_2023_11097_MOESM2_ESM.docx]

**Supplement Fig. 1**

(A) Protein levels of SLC25A32 in LGG and GBM in The Human Protein Atlas.

(B) Representative images of colony formation assays for GBM cells LN229 transfected with si-NC or si-SLC25A32. Cells were seeded at 1000 cells/well, cultured for 2 weeks, fixed and stained with crystal violet, and quantified.

(C) Representative images of colony formation assays for GBM cells GBM#BG5 transfected with OE-NC or OE-SLC25A32. Cells were seeded at 1000 cells/well, cultured for 2 weeks, fixed and stained with crystal violet, and quantified.

(D) Quantitative statistical analysis of tumor sphere formation of GBM cells GBM#P3 transfected with si-NC or si-SLC25A32 and GBM cells GBM#BG5 transfected with OE-NC or OE-SLC25A32.

(E) CCK8 assays showing the proliferation ability of GBM cells GBM#BG5 transfected with OE-NC or OE-SLC25A32 or treated with LY294002.

(F) Fluorescence images and quantification of EdU assays performed to assess the proliferative capacity of GBM cells GBM#BG5 transfected with OE-NC or OE-SLC25A32 or treated with LY294002 (scale bar, 100 μm).
